# Comparative Study of the Safety and Efficacy of Intramuscular Dexamethasone, Betamethasone Phosphate, and Standard Management Protocol in Early-Term Scheduled Caesarean Delivery

**DOI:** 10.7759/cureus.69720

**Published:** 2024-09-19

**Authors:** Namita Gupta, Rajasri G Yaliwal, Subhashchandra Mudanur, Shreedevi Kori

**Affiliations:** 1 Obstetrics and Gynaecology, Shri BM Patil Medical College Hospital and Research Centre, Bijapur Lingayat District Educational Association (BLDE) (Deemed to be University), Vijayapura, IND

**Keywords:** betamethasone phosphate, dexamethasone, early term gestation, respiratory distress syndrome, scheduled cesarean delivery, transient tachypnoea of newborn

## Abstract

Background

Babies born between 37 weeks +0 days and 38 weeks +6 days by scheduled caesarean delivery before the onset of labour are more prone to developing respiratory complications than babies delivered between 39 weeks +0 days and 40 weeks +0 days. Antenatal corticosteroids have been used in preterm births for lung maturity. The advantages of administering antenatal corticosteroids before a scheduled caesarean delivery in the early term remain a subject of debate. While some studies have reported benefits, including a reduction in respiratory issues, the evidence is still inconclusive. The study’s main objective is to compare the efficacy and safety of intramuscular dexamethasone and betamethasone phosphate with standard treatment protocols in early-term infants.

Methodology

A total of 241 pregnant women scheduled for caesarean delivery were screened for eligibility to participate in the study. Out of these, 192 women met the inclusion criteria and were enrolled in the study after providing written informed consent; they were randomised into three groups, with 64 in each group, and were given either betamethasone phosphate or dexamethasone intramuscularly or were considered under standard management protocol (no administration of corticosteroids). The primary comparison was done to observe the development of respiratory distress syndrome, transient tachypnoea in newborns, and the need for neonatal intensive care unit (NICU) admission.

Results

Among 192 pregnant females observed, the incidence of development of respiratory distress syndrome did not differ significantly between the betamethasone, dexamethasone, and standard management groups, with rates of 6.25%, 7.81%, and 4.7%, respectively. Additionally, out of the 192 infants, 15 required neonatal intensive care, including 6 (9.4%) from the betamethasone group, 5 (7.8%) from the dexamethasone group, and 4 (6.3%) from the standard management group. All of these infants had a maximum stay of four days in the NICU, did not require mechanical support, and improved with oxygen therapy.

Conclusion

In our study, in early-term gestation, both the corticosteroid groups showed similar effects to those of the standard management group in reducing neonatal morbidity, with no significant statistical difference. Hence, the choice lies with the treating obstetrician to consider the administration of corticosteroids in early-term caesarean deliveries.

## Introduction

Scheduled caesarean delivery is a planned procedure performed before the actual onset of labour, and it is associated with an increased risk of iatrogenic neonatal respiratory morbidity [1]. Studies have shown that compared to vaginal deliveries, caesarean sections have a higher risk of neonatal respiratory distress syndrome (RDS) and transient tachypnoea of the newborn. Consequently, neonatal respiratory morbidity is a significant concern in scheduled caesarean deliveries [2]. For instance, a study conducted by Kim et al. revealed that caesarean deliveries had a 72.5% incidence of RDS, which is 15 times higher than the 27.5% incidence observed in vaginal deliveries [3].

RDS primarily affects preterm infants, with 93% of those born before 28 weeks of gestation experiencing this condition. Additionally, nearly 10% of late preterm infants and about 1% of full-term infants are affected by RDS. This syndrome is most commonly observed within the first hour after birth [4]. Other complications that may arise include transient tachypnoea of the newborn, neonatal sepsis, and the need for neonatal intensive care unit (NICU) admission [5]. Consequently, the gestation period selected for scheduled caesarean delivery holds significant importance. Respiratory distress is primarily caused by a deficiency of a surfactant, a substance crucial for proper lung development [6,7]. 

Antenatal corticosteroids have drastically changed foetal lung maturation by facilitating surfactant production and improving gaseous exchange and lung compliance. Corticosteroids aid in reducing respiratory distress and transient tachypnoea by enhancing the absorption of fluids in the foetal lungs. This is achieved by increasing the expression of genes responsible for epithelial sodium channels, facilitating fluid absorption and leading to decreased respiratory distress, reduced need for oxygen support, and a shorter duration of NICU stays. However, it is important to note that betamethasone is known to cause hypoglycaemia, whereas dexamethasone can cause neurophysiological defects [8].

The corticosteroids commonly used antenatally for lung maturation are betamethasone phosphate and dexamethasone. The standard regimen for betamethasone is two doses of 12 mg administered intramuscularly, 24 hours apart, whereas dexamethasone is given as four doses of 6 mg each, administered intramuscularly 12 hours apart [9].

Recent studies have found that even full-term neonates can experience respiratory distress. Full term is further classified into the early term (37 weeks + 0 days to 38 weeks + 6 days) and late term (beyond 39 weeks). The incidence of respiratory distress decreases in late-term infants with increasing gestational age: 73.8 per 1,000 at 37 weeks +6 days, 42.3 per 1,000 at 38 weeks +6 days, and 17.6 per 1,000 at 39 weeks +6 days [10,11]. According to the American College of Obstetrics and Gynaecology (ACOG) guidelines, corticosteroids can be administered from 24 weeks +0 days to 33 weeks +6 days of gestation [12]. In contrast, the Royal College of Obstetrics and Gynaecology (RCOG) green top guidelines recommend the administration of corticosteroids for planned caesarean sections between 37 weeks +0 days and 38 weeks +6 days [13]. Therefore, thorough research is necessary to prevent neonatal respiratory distress during this critical gestation period.

This study aimed to compare the safety and efficacy of intramuscular dexamethasone and betamethasone phosphate with the standard treatment protocol in early-term infants, focussing on the development of respiratory morbidity and maternal outcomes.

## Materials and methods

This study was conducted in the Department of Obstetrics and Gynaecology, BLDE (Deemed to be University), Shri B.M. Patil Medical College, Hospital and Research Centre, Vijayapura, Karnataka, India. The participants included consenting pregnant women aged between 18 and 40 years with a gestational age ranging from 37 weeks +0 days to 38 weeks +6 days who were scheduled for caesarean delivery. Exclusion criteria were established for patients with contraindications to dexamethasone and betamethasone, non-reassuring foetal status, or any associated maternal medical conditions, such as septicaemia, maternal diabetes, chronic hypertension, chronic liver or renal disease.

Ethical clearance was obtained from the Institutional Ethics Committee (Reference No. BLDE(D.U.)/IEC/766/2022-23) to conduct the study. The study was registered with the Clinical Trials of India (CTRI/2022/12/048113).

Sample size

The total sample size is 192. With the anticipated proportion of RDS in betamethasone and dexamethasone at 0.4% and 3% [14], respectively, the study required a sample size of 64 per group (i.e., a total sample size of 192 assuming equal group sizes) to achieve a power of 98% for detecting a difference in proportions between betamethasone, dexamethasone, and standard protocol groups at a two-sided p-value of 0.05 with effect size 0.189 using G* power software 3.1.9.73 (Heinrich Heine University Düsseldorf, Düsseldorf, Germany).

Statistical analysis

Data were recorded in a Microsoft Excel spreadsheet (Microsoft® Corp., Redmond, WA) and analysed using the Statistical Package for the Social Sciences (Version 20, IBM Corp., Armonk, NY). Results were presented as mean ± standard deviation (SD) for normally distributed continuous variables or median with interquartile range (IQR) for non‑normally distributed data. Frequency counts were also used for categorical data. A one-way analysis of variance (ANOVA) was used to compare normally distributed continuous variables across three groups. The chi-square test was employed to compare categorical variables between the two groups. A p-value of less than 0.05 was considered statistically significant.

Study design

This study was conducted as a three-arm randomized parallel-group trial. The division of 192 participants into three groups of 64 participants in each group was done by using a computer-based randomization chart from www.randomizer.org, ensuring that participants were assigned randomly to one of the three groups.

Data collection

A total of 241 pregnant women scheduled for caesarean delivery at BLDE (Deemed to be University) Shri B. M. Patil Medical College, Hospital and Research Centre were screened for eligibility to participate in the study. Out of these, 192 women met the inclusion criteria and were enrolled in the study after providing written informed consent.

A computer-generated randomization chart divided the participants into three groups, each consisting of 64 pregnant women. It is a single-blinded study where participants were unaware of the treatment they would receive at the time of consent, but the treating obstetrician was aware of the patient group allocation.

Women assigned to the first group received three doses of dexamethasone, 8 mg each, administered intramuscularly, 8 hours apart over 24 hours. Women assigned to the second group received two doses of betamethasone phosphate, 12 mg each, administered 12 hours apart within 24 hours. Women assigned to the third group were part of the standard management protocol and were not given any corticosteroid treatment.

Neonates born to these women were assessed based on the respiratory rate, APGAR scores at 1, 5, and 10 minutes, heart rate, the need for NICU admission, and the duration of the NICU stay. Additionally, treatment efficacy was evaluated regarding the development of respiratory distress syndrome and transient tachypnoea in the newborn.

## Results

**Figure 1:**
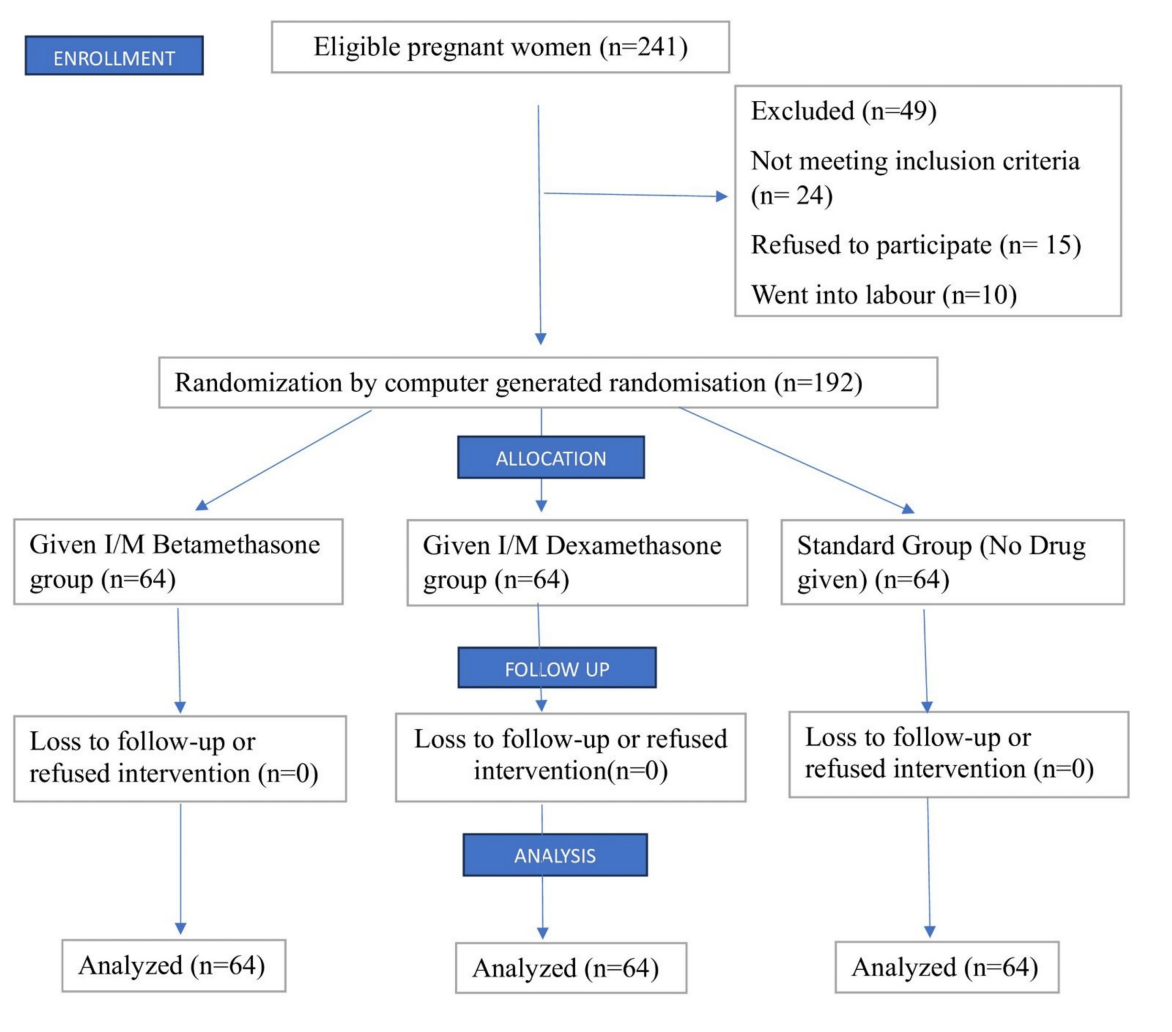
Consort flow diagram

During the study period, a total of 241 pregnant women scheduled for caesarean delivery were considered for inclusion. Out of these, 192 women met the inclusion criteria and were randomised into groups I, II, or III, as shown in Figure 1.

Table 1 describes the baseline parameters, such as the age of the pregnant women and gestational age, and shows a statistically insignificant p-value. In our study, both the betamethasone and standard group (no drug) majority belonged to 20-24 years of age, whereas in the dexamethasone group, the majority belonged to the 25-29 age group (p-value of 0.353). Also, from 37 weeks + 0 days to 37 weeks + 6 days of gestation, we had 51.56%, 40.63%, and 40.63% cases in the betamethasone group, dexamethasone group and standard (no drug) group. 38 weeks + 0 day to 38 weeks + 6 days of gestation, we had 48.44%, 59.38%, and 59.38% cases in the betamethasone group, dexamethasone group, and standard (no drug) group.

**Table 1:** Baseline parameters in three groups *Significant p-value is <0.05

Characteristics	Betamethasone group (n=64)	Dexamethasone group (n=64)	Standard group (no drug) (n=64)	p-value
Age group (year)				
<20	2(3.1%)	0(0%)	1(1.6%)	0.353
20–24	28(43.8%)	23(35.9%)	29(45.3%)	
25–29	20(31.3%)	33(51.6%)	23(35.9%)	
30–34	12(18.8%)	6(9.4%)	8(12.5%)	
>35	2(3.1%)	2(3.1%)	3(4.7%)	
Gestational age (weeks)				
37 weeks + 0 days to 37 weeks + 6 days	33(51.56%)	26(40.63%)	26(40.63%)	0.23
38 weeks + 0 days to 38 weeks + 6 days	31(48.44%)	38(59.38%)	38(59.38%)	

Table 2 represents the parameters used for assessing neonatal outcomes like neonate heart rate, respiratory rate, and APGAR score at 1, 5, and 10 minutes, followed by the requirement of NICU admission along with the duration of stay and treatment modalities considered for neonates. It was observed that in the present study, nearly 18 participants (28.1%) in the betamethasone group, 15 participants (23.4%) in the dexamethasone group, and 14 participants (21.9%) in the standard group exhibited a heart rate of less than 140 bpm. Additionally, three participants (4.7%) from the betamethasone group, one participant (1.6%) from the dexamethasone group, and three participants (4.7%) from the standard group had a heart rate of more than 160 bpm, which were not statistically significant, as indicated by the p-value.

**Table 2:** Neonatal outcome for betamethasone, dexamethasone versus standard (no drug) group *Significant p-value is <0.05. NICU: neonate intensive care unit; APGAR: Appearance, Pulse, Grimace, Activity and Respiration; RDS: respiratory distress syndrome.

Character	Betamethasone group			Dexamethasone group			Standard group (no drug)			p-value
	N (%)	Mean	Standard deviation	N (%)	Mean	Standard deviation	N (%)	Mean	Standard deviation	
Neonate heart rate (bpm)										
<140	18(28.1)	-	-	15(23.4)	-	-	14(21.9)	-	-	0.966
140–149	40(62.5)	-	-	39(60.9)	-	-	47(73.4)	-	-	
150–159	3(4.7)	-	-	9(14.1)	-	-	0(0)	-	-	
>160	3(4.7)	-	-	1(1.6)	-	-	3(4.7)	-	-	
Neonate respiratory rate (cpm)	-	26.75	5.23	-	26.69	4.69	-	26.31	4.69	0.086
APGAR score at										
One minute	-	7.81	0.47	-	7.91	0.47	-	7.88	0.42	0.403
Five minutes	-	8.27	0.51	-	8.44	0.50	-	8.39	0.61	
10 minutes	-	8.83	0.38	-	8.94	0.24	-	8.89	0.36	
NICU admission										
Yes	6(9.4)	-	-	5(7.8)	-	-	4(6.3)	-	-	0.86
No	58(90.6)	-	-	59(92.2)	-	-	60(93.7)	-	-	
Duration of NICU stay										
0 day	58(90.63)	-	-	59(92.19)	-	-	60(93.75)	-	-	0.06
One day	4(6.25)	-	-	2(3.13)	-	-	0(0)	-	-	
Two days	0(0)	-	-	1(1.56)	-	-	2(3.13)	-	-	
Three days	2(3.13)	-	-	0(0)	-	-	2(3.13)	-	-	
Four days	0(0)	-	-	2(3.13)	-	-	0(0)	-	-	
Treatment given to neonates										
No	59(92.2)	-	-	59(92.2)	-	-	60(93.8)	-	-	0.86
Nasal prongs	2(3.1)	-	-	2(3.1)	-	-	2(3.1)	-	-	
Oxygen hood	1(1.6)	-	-	1(1.6)	-	-	0(0)	-	-	
Room air	2(3.1)	-	-	2(3.1)	-	-	2(3.1)	-	-	
Development of RDS	4(6.25)	-	-	5(7.81)	-	-	3(4.7)	-	-	0.81

The one-way ANOVA method was used to calculate the mean respiratory rate and APGAR score at 1, 5, and 10 minutes in all three groups, which is considered a parameter for the calculation of the development of respiratory distress syndrome and the need for neonatal resuscitation. The mean respiratory rates in betamethasone, dexamethasone, and standard (no drug) groups are 26.75, 26.69, and 26.31, respectively, with no significant p-value. The APGAR score at 1, 5, and 10 minutes in the betamethasone group was 7.81, 8.27, and 8.83, whereas, in the dexamethasone group, it was 7.91, 8.44, and 8.94, respectively, with no significant difference statistically.

The above table also shows that a total of 15 NICU admissions were recorded across all three groups, with 6 admissions (9.4%) from the betamethasone group, 5 admissions (7.8%) from the dexamethasone group, and 4 admissions (6.3%) from the standard group. The differences in NICU admission rates among the groups were not statistically significant. In our study, the development of respiratory distress syndrome was seen in 4 (6.25%) betamethasone group, 5 (7.81%) in the dexamethasone group, and 3 (4.7%) in the standard (no drug) group with no statistically significant difference.

The study revealed that both the betamethasone and standard groups had a minimum NICU stay of three days, whereas the dexamethasone group had a four-day NICU stay. However, the difference in the duration of NICU stays among these groups was not statistically significant (p-value = 0.06). Oxygen therapy was administered in betamethasone and dexamethasone groups among the 15 neonates admitted to the NICU. However, no statistically significant difference was found between the two groups regarding oxygen therapy usage.

## Discussion

This study assessed the safety and efficacy of intramuscular dexamethasone versus intramuscular betamethasone phosphate and standard management (no drug) in antenatal women who visited the hospital for early-term scheduled delivery. The safety and efficacy of these interventions were assessed concerning the development of respiratory distress syndrome, transient tachypnoea of the newborn, and the need for NICU admission.

The study found no significant difference among the three groups regarding the development of respiratory distress syndrome. The mean respiratory rate across the three groups was 26 cycles per minute, and the incidence of transient tachypnoea in the newborn was similar among the dexamethasone, betamethasone, and standard groups, with rates of 98.4%, 95.3%, and 95.3%, respectively.

NICU admissions were observed in 15 out of 192 neonates: 6 (9.4%) from the betamethasone group, 5 (7.8%) from the dexamethasone group, and 4 (6.3%) from the standard management group. The differences among the three groups were statistically insignificant, with a p-value of 0.86. In contrast, other studies have shown significant differences. For example, a study by Issa et al. was done between the betamethasone and dexamethasone groups and found that 5 (2.1%) cases in the betamethasone group and 25 (10.6%) cases in the dexamethasone group had NICU admission with a significant p-value (<0.001) [14]. Li et al. considered two groups by giving or not giving corticosteroids and observed that 190 (76.9%) cases from the corticosteroid group and 71 (75.5%) from the no corticosteroid group had NICU admission with a statistically significant difference (p-value = 0.043) [15].

Various studies have investigated the impact of corticosteroids on NICU stay duration, revealing a range of findings with differing levels of statistical significance. Gupta et al., where there was a comparison between the corticosteroid (36.4%) and the non-corticosteroid (18.8%) groups, had insignificant differences with a p-value of 0.054 [16]. Khushdil et al. compared the corticosteroid group where dexamethasone (2.5%) was given and no corticosteroid group (7.79%) had NICU admission with an insignificant difference (p-value = 0.007) [17].

Further assessment of NICU stay duration revealed that in a sample of 15 cases, the dexamethasone group had a maximum stay of four days (3.13%), whereas both the betamethasone group (3.13%) and the standard group had a stay of three days (3.13%). This difference was not statistically significant, with a p-value of 0.06.

Issa et al. comparison between the two corticosteroids was significant with a p-value of 0.011, with the betamethasone group having a two- to three-day stay and the dexamethasone group having a four- to five-day stay [14]. Arsad et al. found no statistically significant difference in the average duration of NICU stay, with the dexamethasone group averaging one day and the non‑corticosteroid group averaging five days, with a p-value of 0.27 [2].

Dileep et al. reported that the dexamethasone group had an average stay of three to four days, while the non-corticosteroid group had an average stay of two to three days, with an insignificant p-value of 0.18 [10]. Li et al. study had nine cases with 3-31 days of stay in the standard groups, whereas, after administration of corticosteroids, nine cases were admitted for less than two days, 10 cases for two to seven days, and 11 cases for more than seven days with an insignificant p-value of 0.197 [15].

In the study by Gupta et al., the corticosteroid group had two days of stay, whereas the non-corticosteroid group also had a minimum of three days of NICU stay with an insignificant p-value of 0.769 [16]. In the study by Khushdil et al., in the corticosteroid group, nearly 233 (97.08%) had less than six days stay, 3 (1.25%) cases had 6-24 days stay, and only 1 (0.41%) case had 24-48 days stay, whereas in the non-corticosteroid group, nearly 275 (93.22%) had less than six days stay, 4 (1.35%) case had 6-24 days stay, and 5 (1.69%) cases had 24-48 days stay with an insignificant p-value of 0.154 [17].

Another study by Elbohoty et al. had a minimum of seven days stay in either of the groups (dexamethasone versus standard group) with an insignificant p-value of 0.342 [18]. Al Riyami et al. observed that patients receiving corticosteroids had a NICU stay of four to seven days, while those not receiving corticosteroids had a NICU stay of three to five days, with no statistical significance (p-value = 0.113) [19].

Study limitation

One limitation of this study is its small sample size, which may affect the generalisability and robustness of the findings.

Study strength

Despite this constraint, the study exhibits notable strengths. This study considered three groups in parallel, facilitating a comprehensive evaluation of safety and efficacy in developing neonatal morbidities. This comparative approach enhances the study’s capacity to assess and understand the nuances of neonatal health outcomes effectively.

## Conclusions

Respiratory distress syndrome is the most prevalent neonatal complication observed after delivery. To address this issue, antenatal corticosteroids such as betamethasone and dexamethasone have been used to mitigate neonatal distress. The current study examined the effects of these corticosteroids administered during early-term gestation. Both corticosteroid treatment groups showed similar outcomes in reducing neonatal morbidity compared to the standard management group, with no statistically significant differences observed. Consequently, the decision to administer corticosteroids in early-term caesarean deliveries should be guided by the treating obstetrician based on individual clinical considerations.
